# Parasitological Indices of Malaria Transmission in Children under Fifteen Years in Two Ecoepidemiological Zones in Southwestern Burkina Faso

**DOI:** 10.1155/2017/1507829

**Published:** 2017-02-13

**Authors:** Aristide S. Hien, Ibrahim Sangaré, Sanata Coulibaly, Moussa Namountougou, Léa Paré-Toé, Anicet Georges Ouédraogo, Abdoulaye Diabaté, Brian D. Foy, Roch K. Dabiré

**Affiliations:** ^1^IRSS/Centre Muraz, Bobo-Dioulasso, Burkina Faso; ^2^Université Polytechnique de Bobo-Dioulasso, Bobo-Dioulasso, Burkina Faso; ^3^Colorado State University, Fort Collins, CO, USA

## Abstract

Twenty years after the latest publications performed on the parasitological indices of malaria transmission in northwest of the second city of Burkina Faso, it was important to update the epidemiological profile of malaria in children under the age of 15 years. The objective of this study was to determine and compare the parasitological parameters of malaria transmission by season, area, and age in the two zones (rice and savanna) in the northwest of Bobo-Dioulasso, Burkina Faso. Overall, the results showed that there was no significant difference in the parasitological indices of malaria transmission within children under fifteen years between the rice site and the savannah site and whatever the season (*P* > 0.05). The profound environmental modifications that occurred in the rice zone would have led to changes in vector behavior and consequently to changes in the epidemiological profile of malaria, contrary to the results obtained since the last publications. An entomological study correlated with this study is therefore necessary for effective decision-making for the malaria control in both areas. Future research must now focus on the impact that these profound environmental modifications of rice area are having on malaria control in Burkina Faso.

## 1. Background

Febrile illness is the most common and important component of malaria syndrome in sub-Saharan Africa [1]. Children under 5 years of age and pregnant women constitute the major groups for developing life-threatening malaria [2]. In Burkina Faso, malaria is both endemic and hyperendemic [2] and remains a major public health problem. Malaria control policies in Burkina Faso include free distribution of insecticide-treated bed nets (ITNs), Intermittent Preventive Treatment (IPT) for pregnant women, and treatment of malaria cases with artemisinin-based combination therapy (ACT). Seasonal Malaria Chemoprevention (SMC) was adopted as policy in 2012 according to the WHO recommendations and a national plan for introducing SMC has been implemented because clinical trials in Mali and Burkina Faso have shown a strong protective effect of SMC against clinical malaria and severe malaria even in areas with high net coverage [3]. But despite recent efforts in scaling up these interventions, coverage of ITNs and access to treatment with ACTs remain relatively low [4].

Furthermore, Burkina Faso, like many other African countries, is also facing major food security challenges. The solution to this challenge has been a policy to promote the development of irrigated crops including rice and is based on water management and the development of irrigation schemes. Although hydroagricultural works can provide enormous benefits, it is also acknowledged that population health can be negatively influenced by them through profound changes in the ecosystem. Indeed, irrigation promotes the proliferation of mosquito breeding sites and affects both the duration and speed of replication of* Anopheles* mosquitoes. The landscaped perimeter of “Vallée du Kou” was realised within the framework of cooperation between the Volta (today Burkina Faso) and China in 1969 and construction began in 1970 on 1000 hectares. This perimeter led to health consequences by promoting the proliferation of* Anopheles* mosquitoes; in particular, the density of* Anopheles gambiae*, the major vector of malaria transmission in the area, multiplied 20-fold and this was followed by a doubling of the rate of malaria transmission [5]. The annual number of infectious bites has increased significantly in the valley compared to the neighboring savannah [5, 6].

However, while past results have been focused on the evaluation of entomological parameters of malaria transmission, few studies have focused on the evaluation of parasitological parameters of malaria transmission in both the rice-growing area and the surrounding savannah.

Twenty years have passed since the last publications were published on the parasitological indices of malaria transmission in the area [7, 8]. This study was performed to update our knowledge of parasitological indices in the area. There are continuing questions about whether irrigated perimeters are more or less suitable to malaria compared to the savanna area and if parasitological indices are higher or lower in the rice-growing area than in the savanna area. This study was initiated to answer these questions and provide new information to update the data on the parasitological parameters of malaria transmission in these two different ecoepidemiological areas with a focus on the most vulnerable group of the community, children under fifteen years. Our objective was to determine and compare the parasitological parameters of malaria transmission by season, area, and age range in both zones (rice and savanna) in northern-west Bobo-Dioulasso, Burkina Faso.

## 2. Methods

### 2.1. Study Area

**Figure 1:**
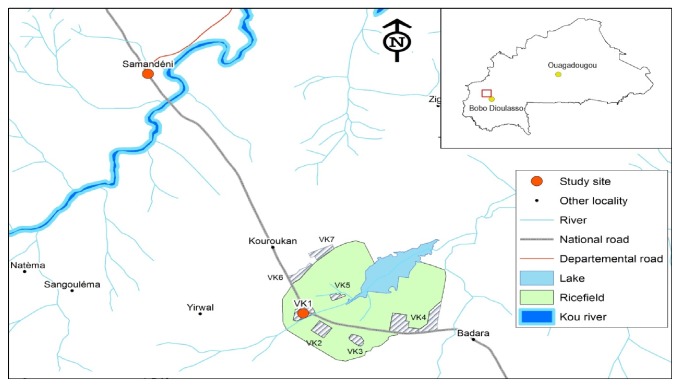
Location of study sites. Colored red points represent the two study sites located on the Bobo-Faramana-Mopti road colored in black. VK1 is the study site in rice area and Samandeni is study site in savannah area. The plot colored green shows the rice field with different rice-growing sites (VK1–VK7).

The District of Dandé is located in the northern-west of Bobo-Dioulasso (Figure 1). The climat is of Sudano-Guinean type, alternating with a wet season and a dry season unevenly distributed throughout the year. The dry season occurs from November to June and the rainy season from June to October with peak maximum rainfall in August-September. The average annual rainfall is about 1000 mm. April is the hottest month (39°C) and December is the coldest month (23°C). The minimum and maximum temperatures range from 20°C to 27 (average 23°C) and 33°C to 39°C (average 37°C), respectively. The rainy season is the most suitable for mosquito breeding and transmission of malaria in the area, which extends from June to November, but residual malaria transmission can last beyond this period in some places.* An. gambiae* s.l. is the major vector of malaria in the study area, with* Anopheles gambiae* predominating in some places while* Anopheles coluzzii* is predominant in the rice-growing area;* An. funestus* and* An. arabiensis* are present in small proportions and frequencies increase towards the end of the rainy season [5, 9].

Two localities from this district were retained as our study sites as representative of two ecoepidemiological areas. Study sites in rice and savanna areas are, respectively, the Kou Valley 1 (VK1) (11°22′56, 4′′N; 4°25′13, 1′′W) and Samandeni (11°28′22, 7′′N; 4°28′30, 8′′W). These two localities are separated by 15 km and are located on the Bobo-Faramana-Mopti road. The populations of the two study sites are composed of about fifteen ethnic groups and the main spoken language is Dioula. Populations are engaged in subsistence agriculture in the savanna area; however in the rice area the main activity is the cultivation of rice. According to the data from Dandé District in 2014, the two localities counted about 4018 and 10555 inhabitants in VK1 and Samandeni, respectively. Children and adolescents (0–14 years) represented 45.1% of the population. The prevalence of malaria in children 0 to 14 years old one year before the start of the survey based on rapid diagnostic tests (RDTs) and reported by the respective health facilities was 29.8% in VK1 and 15% and in Samandeni.

### 2.2. Sample Size and Study Populations

We have carried out a random sampling by relying on subjects who were present at the time of our passage in each site until we reached the estimated number of subjects planned for the study. based on a random sampling of 80 to 110 children from 0 to under fifteen per passage, we conducted six passages, three passages in the dry season (November, January, and April) and three passages in the rainy season (June, August, and October) in each study site. The objective was to reach at least 300 children per season and per site required for the study, considering prevalence of 29.8% in the savanna zone and 15% in the rice zone in rainy season with an accuracy of 10%, standard deviation (1.96) corresponding to the risk of error 5% and 10% of nonrespondents.

The study included all children in the village aged between 0 and under 15 years whose parents gave their consent. Those excluded were study children whose parental consent was not obtained and children not residing in the two sites selected. Also, subjects who received antimalarial treatment during the last 14 days are excluded.

The study was conducted for one year, from November 2014 to October 2015.

### 2.3. Thin and Thick Smears

Blood samples were collected monthly by the standard finger-prick method according to the season. The thin and thick blood smears were air dried. Thereafter, the thin and thick smears were fixed in ethanol, then stained with 10% Giemsa (Sigma) in phosphate buffer (pH 7.0) for 30 minutes, and examined using oil immersion magnification (100x). Each slide was examined by two microscopists, who estimated the parasite density by counting the number of asexual* P. falciparum* parasites in fields containing 200 white blood cells and multiplied that number by 25 to estimate the number of asexual* P. falciparum* parasites per *μ*L (based on an average white blood cells count of 8000 per *μ*L [10]). Slides were considered positive or negative after two readers examined fields containing 200 white blood cells. Slides on which there was disagreement were examined by a third microscopist to confirm the result. Random checks were carried out on the slide counts (to include at least 10% of all slides) by independent microscopists to ensure quality control. An individual was considered positive if malaria parasites were detected in the blood smear.

The parasite carriage rate or the plasmodic index (PI) was estimated by the proportion of positive numbers from examined thick smears and the gametocytemic index (GI) by the proportion of subjects presenting sexual forms or gametes in the thick smear. Parasite densities of sexual and asexual forms were expressed as the mean number of parasites per microliter (*μ*L) of blood. Parasite densities obtained by the study site were classified and annotated as follows: 1: less than 1000 parasites/*μ*L; 2: 1,000–5,000 parasites/*μ*L; 3: 5,001–10,000 parasites/*μ*L; and 4: more than 10,000 parasites/*μ*L.

### 2.4. Clinical Examination

Axillary temperatures were measured by skilled health personnel in order to detect any temperature greater than 38°C (the pyrogenic threshold considered in our study). Any child who had fever above 38°C was considered clinically ill if he/she had fever (a core body temperature ≥ 38°C) and malaria parasites identified from the blood smear. Any child that was clinically ill at the survey date was taken to the nearest public health facility for treatment free of charge. The temperature was taken using an electronic thermometer (Ormon^R^).

### 2.5. Ethical Approval

Ethical approval of the study was obtained of ethic committee from Centre Muraz (Bobo-Dioulasso). After its approval, each participant was then informed about the protocol; care was given to all study participants. Oral informed assent was then obtained from each participant. All the study participants considered clinically ill were examined and treated in line with the national malaria guidelines of Ministry of Health, Burkina Faso.

### 2.6. Data Analysis

Excel and GraphPad Prism 5 software were used, respectively, for entry and analysis data. The malariological indices were determined and compared by site, season, and age range to determine the variations between the two ecoepidemiological areas. The nonparametric Wilcoxon signed-rank test was used to compare longitudinal samples throughout season and the Mann–Whitney test for mean comparison between areas with an alpha of 0.05.

## 3. Results

### 3.1. Participant Recruitment

The census of children under fifteen years of selected households in the two study sites counted 1,121 children of both sexes eligible for this study. There were 555 children from VK1 and 566 children from Samandeni. Their distribution according to sex by site ranged from 242 females to 313 males from VK1 and 246 females to 320 males from Samandeni (Table 1). However, the distribution according to age group showed that 36 children (6.48%) in VK1 compared to 31 (5.47%) in Samandeni were aged less than one year; 116 (20.9%) compared to 183 (32.33%) between 1 and 5 years of age; 249 (44.86%) compared to 226 (39.92%) were between 5 and 10 years of age, and 154 (27.74%) compared to 126 (22.26%) were between 10 and 15 years of age. There was no statistically significant difference in the representation of the two sexes and age groups in the study site (*P* > 0.05).

### 3.2. Plasmodic Index by Site

A total of 1121 thick and thin smears were performed during the study period and examined. 558 of them were carriers of malaria parasites and the mean plasmodic index was 49.77% (CI: 34.99–58.01). The plasmodic index was 40.85% (95% CI: 21.89–59.81) and 61.66% (95% CI: 47.55–75.76), respectively, in the rice zone and in the savanna zone. It was higher in the savanna zone and lower in the rice zone, but this difference was not statistically significant (*P* > 0.05).

### 3.3. Seasonal Variations of the Plasmodic Index and* Plasmodium* Species

**Figure 2:**
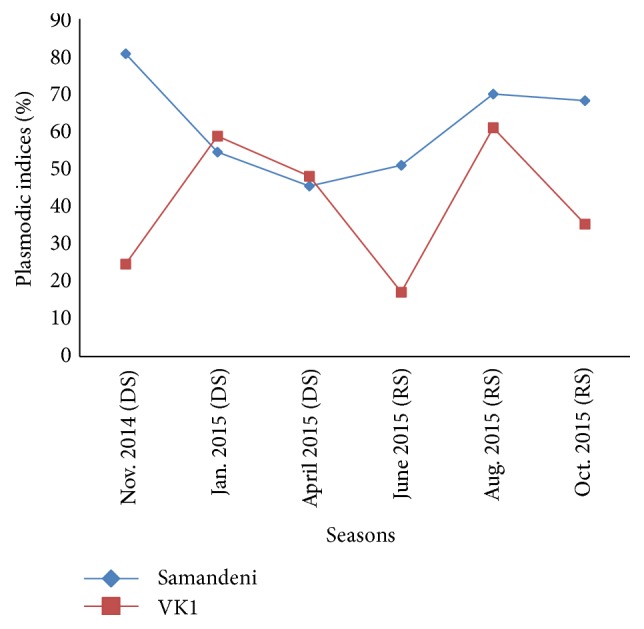
Seasonal variation of plasmodic indices of study sites. DS: dry season; RS: rainy season; the blue curve: seasonal variation of Samandeni children's plasmodic index; the red curve: seasonal variation of the VK1 children's plasmodic index.

Overall, seasonal variations in plasmodic index were not significant in the different ecoepidemiological zones (*P* > 0.05). But it was higher in Samandeni (savanna zone) than in VK1 (rice area) at the beginning of the dry seasons (November 2014: mean PI 80.72% versus 24.69%) and rainy season (June: mean PI 51% versus 17.18%) (*P* < 0.05). However the evolution of the plasmodic index according to the dry season months showed a higher parasitaemia in January and April in the rice zone compared to the savanna zone (*P* > 0.05) (Figure 2).

*Plasmodium falciparum* was the most prevalent in the dry season, as well as in the rainy period, but with a higher proportion in the rainy season whatever the site. Three species of malaria parasites have been observed during the rainy season in the savanna area while two species (*P. falciparum* and* P. malariae*) were detected only during the dry season in the rice zone. Mixed infections (*P. falciparum* and* P. malariae*) were observed in positive slides only during the rainy season in the rice zone and in both seasons in the savanna zone. Three cases of mixed infections were found in the rice area compared to 15 cases in the savannah area. No cases of triparasitism were found in either site.

### 3.4. Variations of the Plasmodic Index according to Age Ranges

**Figure 3:**
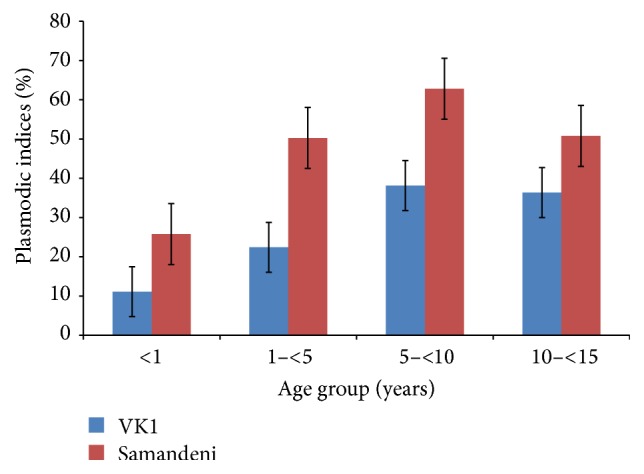
Plasmodic indices with standard errors bars (SEM) according to age (means ± SEM). When standard error bars (SEM) do not overlap, this means that the difference between two means is statistically significant (*P* < 0.05).

The plasmodic index increased with age, as it was very low in toddlers and reached a maximum in children between 5 and 10 years old, but it then decreased in children >10 years old, regardless of the study site. The children between 5 and 10 years old were the most infected and had plasmodic index of 62.33% in Samandeni and 38.15% in VK1 (Figure 3). The least infected children were those <1 year old in VK1 (11%) in contrast to those of Samandeni where a quarter of this age group were infected. The proportion of children aged 2 to 9 had a plasmodic index of 53.48% and 55.6% (Table 1), respectively, in the rice and savannah zone.

For the two biogeographic zones, the plasmodic index showed no significant differences between the different age groups by site (*P* > 0.05) and between the two study sites (*P* = 0.11).

### 3.5. Gametocytemic Index

Of the 551 thick smears examined for the rice-growing area, twenty-one of these showed gametocytes of* Plasmodium falciparum*, thus a gametocytic index of 3.78% (21/551). In the savanna zone, gametocytic index was 1.94% (11/566). This difference in the gametocyte rate found between the two sites was statistically significant (*P* < 0.05). Gametocytes were found in the positive slides, whatever the period and the study site but with a higher proportion in the rainy season in the rice zone (2.70%) compared to the savanna zone (0.5%). The subjects likely to infect vectors became more important in the population during the rainy season in the rice-growing area.

For all 1,121 specimens examined, the mean gametocytemic index was 2.85%.

### 3.6. Distribution of Parasite Densities in the Different Ecoepidemiological Zones

The lowest parasite density was 16 parasites/*µ*L of blood and the highest was 471,889 parasites/*µ*L, all observed in Samandeni during the dry season (November 2014 and January 2015). For a better interpretation of parasite density in order to compare the two study populations, we expressed parasite densities by the geometric mean parasite density (GMPD) in children from 0 to 15 years, taking into consideration the positive and negative slides examined. The difference in geometric mean parasite densities between the rice area (2706; 95% CI: 2441–2970) and the savanna (2742; 95% CI: 2543–2940) was not significant (*P* = 0.68; mean square = 3888, Df = 1). However, geometric means have evolved significantly in each study site (*P* = 0.03). In contrast, they were comparable whatever the season in the two study sites (*P* < 0.05).

Parasite densities less than 1000 parasites/*μ*L of blood were most frequently encountered in children aged 5 to 10 years, 36.75% in Samandeni (208/566) and 22% in VK1 (122/555) at both sites (*P* = 0.01). There were 4% against 5% of subjects, respectively, in rice and savannah areas who had parasite densities above 10,000 parasites/*μ*L.

### 3.7. Relationship between Body Temperature and Infection Prevalence of Malaria

Of a total of 555 children examined in VK1, 217 were carriers of malaria parasites and 140 were febrile (25.22%). Of the 140 participants with fever, 43.57% were carriers of malaria parasites compared to 56.43% who were not carrying* Plasmodium*. This difference is not statistically significant (*P* > 0.05).

The presence of fever in VK1 was not related to the presence of asexual malaria parasites in the blood (OR < 1). Contrary to VK1, the presence of asexual malaria parasites was strongly related to the presence of fever in Samandeni (OR = 28.61 with 95% CI: 3.60–158.80), with 53.07% of participants having both fever and* Plasmodium* compared to 46.92% with fever but no* Plasmodium* (Table 2). In both sites, as age increased, so did the number of participants carrying malaria parasites who also had fever: 0% in children less than one year old, 7.69% for children 1–5 years old, 34.61% for children between 5 and 10 years old, and 57.69% in children 10–15 years old (*P* < 0.001).

The risk of having a febrile attack increased with parasite density group in VK1. This risk became significant (*P* < 0.01) with a parasite density > 10,000 parasites/*µ*L (odds ratio of 1.5 for PD > 10,000; 95% CI: 0.96–5.6). However, there was no variation in the different parasite density groups in Samandeni, but the risk of having a febrile attack was higher in the lowest parasite density group (1000 ≤ DP < 5000).

## 4. Discussion

The assessment of parasite prevalence provides indications on the extent of transmission and the state of circulation of parasites in a community.

The plasmodic index indicates the percentage of subjects carrying* Plasmodium* in the peripheral blood. This index, in subjects less than one year old, may be a good indication of the intensity of transmission. In adolescents and adults, it provides information on the degree of immunity of this population. The mean rates of the plasmodic indices in the two localities were all greater than 10%. This confirms the presence of endemic and stable malaria in two localities. Moreover, at the 3rd African Conference on Malaria, held in Yaoundé, Cameroon, in July 1962, it was decided to retain this terminology (hypo-, meso-, and holoendemic) using the criterion of the plasmodic index in children aged 2 to 9 years. The plasmodic indices (PI) obtained confirm in this study area the presence of hyperendemic malaria according to the plasmodic indices observed in children aged 2 to 9 years in the two study sites.

Stable malaria is referred to when the transmission season is very long and there is little change in incidence during the year and from year to year. In such conditions, climate change is too insignificant to influence anopheline transmission activity, temperature ensures a rapid sporogonic cycle, the vector is highly anthropophilic, and vector survivorship is long. Stable malaria is most often due to* P. falciparum* malaria in Africa, and there is a very high degree of immune protection in the population. In our study, the predominant plasmodial species in both localities was* P. falciparum*. This confirms the literature data that report that this species is most common in West Africa [11–13]. The dominance of this species, according to Gaye et al. [14], is likely due to better efficiency of transmission of its sporozoites. Also, the results of the study remain consistent with this hypothesis, whereby all children carried* Plasmodium* either alone (95%) or in association with* P. malariae* (1–4%) or* P. ovale* (about 1%). These prevalence rates were higher than those of Meetselaar and Van Thiel [11], in the city of Bobo-Dioulasso which is located 25 km from the study area. No infections occurred with all three species together, and no infections with* P. vivax* were observed, which was congruent with historical epidemiological studies [15]. In addition, there was no seasonal variation in the plasmodic indices. They were also comparable in the two study sites. This could be explained by the profound environmental changes in this rice-growing region. In the past in this rice zone, rice was grown according to a well-defined calendar with two growing periods, one in the dry season and the other in the rainy season. The abundance of malaria vectors was also closely linked to this cultural rhythm of rice harvesting. But today it is no longer the case. There is no calendar and therefore vectors can be found at any time of the year as in the savanna zone. This could explain why there is no difference in the evolution of the plasmodic indices and the parasitic loads in the rice zone as in the savanna zone. Instead, they show that plasmodic indices are stable throughout the year in Samandeni but falls into VK1. But this difference in the plasmodic indices was not significant. This suggests that children in the rice area are exposed to malaria in the same way as children in the savanna zone and explains why we did not find any significant difference in the variation of the plasmodic indices between the age groups and the site. This result is the same with the parasite carriage portage. Indeed, the parasitic densities were comparable whatever the time of the year between the two sites. The age group most susceptible to malaria was children aged 5–10 years, in contrast to other studies that have demonstrated the most susceptible children are those less than five years old [15–17]. Indeed, the national malaria control policy of the Burkina Faso promotes the use of insecticide-treated nets among the most vulnerable groups in the community, who are defined to be pregnant women and children under five years of age. This approach has an additional benefit in reducing malaria in this age group in these study sites. The fact that children aged 5 to 10 years are not receiving these needed benefits as those less than one year could lead to high exposure to mosquito bites in both localities. This could contribute to increased parasite densities in this age range. Moreover, our study found a higher gametocytemic index in VK1 (3.78%) than in the savannah zone (1.94%). The profound environmental changes observed in the rice area could explain these results. Ecological changes that occurred in the rice-growing areas are one possible reason for the strong gametocytemic index that was observed. This would help to maintain permanent transmission until the dry season, as shown by the results of the study.

## 5. Conclusion

This study investigated the epidemiological profile of malaria in ecologically different environments in children under 15 years in Burkina Faso. In Burkina Faso, as in most of the developing countries, food security is crucial and requires water storage and management. The intensification of irrigated crops is a response to this challenge. Our study suggests that hydroagricultural developments in the northwest of the city of Bobo-Dioulasso influences malaria prevalence and morbidity rates in the region. Indeed, the morbidity rate was comparable between rice area and savanna area. We think that the development of irrigated crops, in particular rice cultivation in Sudan zones as in Burkina Faso, has encouraged the proliferation of mosquitoes, some of which are malaria vectors which would lead to increased number of malaria cases. The once stated theory of the “paradox of Vallée du Kou” seems to have changed over time, and now the rice cultivation zone has similar ecoepidemiological characteristics of the nearby savannah zone.

## Figures and Tables

**Table 1:** Study participant's characteristics.

Characteristic	Rice site	Savanna site	*P* value
Sex			
Male	242 (43.6)	246 (43.5)	>0.05
Female	313 (56.4)	320 (56.5)	>0.05

Age			
Mean ± SD	8.625 ± 2.905	6.804 ± 4.242	0.06
Range	1–14.8	0.8–14.9	

Age group			
<1 year	36	31	>0.05
1–<5 years	116	183	
5–<10 years	249	226	
10–<15 years	154	126	
2–9 years	285	329	

Temperature (°C)			
Mean ± SD	36.41 ± 0.7646	36.68 ± 0.6816	0.0786
Range	34.5–37.60	35–39.20	

Plasmodic index			
Mean ± SD	40.85 ± 18.07	61.66 ± 13.44	0.0931
Range	17.18–61	45.45–80.72	
2–9 years	53.48%	55.6%	

GMPD			
Mean ± SD	2706 ± 252	2742 ± 189	0.93
Range	2335–3000	2414–2912	

SD: standard deviation. GMPD: geometric mean parasite density and numbers in brackets represent percentages. There were no significant differences between participant's characteristics of two study sites.

**Table 2:** Relation between presence of fever and malaria-carrying parasite.

Parasitemia	Status of fever		Total	Odds ratio
	No fever	Fever		
VK1				
Positive thick smears	151	61	217	0.93 0 ≤ OR ≤ 2.1
Negative thick smears	257	79	338	
Total	408	140	555	

Samandeni				
Positive thick smears	241	69	310	28.61 3.60 ≤ OR ≤ 158.8
Negative thick smears	195	61	256	
Total	436	130	566	

## References

[1] Greenwood B. M., Bojang K., Whitty C. J. M., Targett G. A. T. (2005). Malaria. *The Lancet*.

[2] World Malaria Report 2015, http://www.who.int/iris//bitstream/10665/200018/1/9789241565158_eng.pdf

[3] Wilson A. L. (2011). A systematic review and meta-analysis of the efficacy and safety of intermittent preventive treatment of malaria in children (IPTc). *PLoS ONE*.

[4] De Allegri M., Louis V. R., Tiendrébeogo J. (2013). Moving towards universal coverage with malaria control interventions: achievements and challenges in rural Burkina Faso. *International Journal of Health Planning and Management*.

[5] Baldet T., Diabaté A., Guiguemdé T. R. (2003). Etude de la transmission du paludisme en 1999 dans la zone rizicole de la Vallée du Kou (Bama) Burkina Faso. *Cahiers Santé*.

[6] Diabaté A. (2003). *Paludisme au Burkina Faso: Etude de la transmission et répartition géographique de la résistance d'Anopheles gambiae sl. aux pyréthrinoïdes [Ph.D. thesis]*.

[7] Robert V., Gazin P., Boudin C., Molez J. F., Ouedraogo V., Carnevale P. (1985). The transmission of malaria in a wooded savannah area and a rice-growing area around Bobo-Dioulasso (Burkina Faso). *Annales de la Societe Belge de Medecine Tropicale*.

[8] Gazin P., Robert V., Carnevale P. (1985). Longitudinal study of malaria indices in 2 villages of the Bobo Dioulasso region (Burkina Faso). *Annales de la Societe Belge de Medecine Tropicale*.

[9] Dabiré K. R., Diabaté A., Djogbenou L. (2008). Dynamics of multiple insecticide resistance in the malaria vector *Anopheles gambiae* in a rice growing area in South-Western Burkina Faso. *Malaria Journal*.

[10] Payne D. (1988). Use and limitations of light microscopy for diagnosing malaria at the primary health care level. *Bulletin of the World Health Organization*.

[11] Meetselaar D., Van Thiel P. H. (1959). Classification of malaria. *Tropical and Geographical Medicine*.

[12] Gazin P., Robert V., Carnevale P. (1985). Longitudinal study of malaria indices in 2 villages of the Bobo Dioulasso region (Burkina Faso). *Annales de la Societe Belge de Medecine Tropicale*.

[13] Benasseni R., Gazin P., Carnevale P., Boudon D. (1987). Le paludisme urbain a Bobo Dioulasso (Burkina Faso) Etude de la morbidité palustre. *Cahiers ORSTOM Série Entomologie Médicale et Parasitologie*.

[14] Gaye O., Bah I. B., Bengue E., Diallo S., Faye O. (1989). Morbidité palustre en milieu urbain. Etude de 353 accèsfébriles. *Medecine Tropicale*.

[15] Burkot T. R., Graves P. M., Cattan J. A., Wirtz R. A., Gibson F. D. (1987). The efficiency of sporozoite transmission in the human malarias, *Plasmodium falciparum* and *Plasmodium vivax*. *Bulletin of the World Health Organization*.

[16] Baldé M. C., Camara M., Barry A. O. (2001). Etude de la prévalence du paludisme dans 24 villages de la Guinée. *Bulletin de la Société de Pathologie Exotique*.

[17] Olasehinde G. I., Ajay A. A., Taiwo S. O., Adekeye B. T., Adeyeba O. A. (2010). Prevalence and management of Falciparium malaria among infants and children in Ota, Ogun state, Southwestern Nigeria. *African Journal of Clinical and Experimental Microbiology*.

